# Impact on survival of tobacco smoking for cases with oropharyngeal squamous cell carcinoma and known human papillomavirus and p16-status: a multicenter retrospective study

**DOI:** 10.18632/oncotarget.27079

**Published:** 2019-07-23

**Authors:** Christian Grønhøj, Jakob Schmidt Jensen, Steffen Wagner, Christian Dehlendorff, Jeppe Friborg, Elo Andersen, Claus Wittekindt, Nora Würdemann, Shachi Jenny Sharma, Stefan Gattenlöhner, Jens Peter Klussmann, Christian von Buchwald

**Affiliations:** ^1^Department of Otorhinolaryngology, Head and Neck Surgery and Audiology, Rigshospitalet, University of Copenhagen, Copenhagen, Denmark; ^2^Department of Otorhinolaryngology, Head and Neck Surgery, University of Giessen, Giessen, Germany; ^3^Department of Statistics and Pharmacoepidemiology, Danish Cancer Society Research Center, Copenhagen, Denmark; ^4^Department of Oncology, Rigshospitalet, University of Copenhagen, Copenhagen, Denmark; ^5^Department of Oncology, Herlev Hospital, University of Copenhagen, Copenhagen, Denmark; ^6^Department of Otorhinolaryngology, Head and Neck Surgery, Medical Faculty, University of Cologne, Cologne, Germany; ^7^Department of Pathology, University of Giessen, Giessen, Germany

**Keywords:** oropharyngeal cancer, human papillomavirus, smoking, survival

## Abstract

**Background:**

Human papilloma virus (HPV) and tobacco smoking are important risk factors for development of oropharyngeal squamous cell carcinoma (OPSCC).

**Aims/objectives:**

To evaluate the impact of tobacco smoking on survival for cases with OPSCC with known HPV- and p16INK4A(p16)-status.

**Materials and Methods:**

OPSCC cases at the University Hospital of Copenhagen, Rigshospitalet, Denmark (2000–2014) and at University Hospital of Giessen, Germany (2000–2009) were included. Survival was illustrated with Kaplan-Meier plots. The effect of smoking exposure on survival was evaluated by Cox-regression models. HPV-positivity was defined as positivity for both HPV-DNA and p16.

**Results:**

We included 1316 OPSCC cases from 2000–2014 (48% HPV-positive). Smokers had a poorer outcome compared to non-smokers. Considering continuous smoking exposure, adding 10 pack-years of smoking increased hazard ratios irrespective of HPV-status.

We observed a tendency to a greater impact on survival for cases with HPV-neg. tumours compared to cases with HPV-pos. tumours at low numbers of pack-years, yet the survival was similar at high numbers of pack-years. There was no significant difference in the impact of HPV-status on survival for non-smokers, however a highly significant difference for smokers.

**Conclusions and Significance:**

Smoking-status and number of pack-years at time of diagnosis impact survival for cases with OPSCC independent of HPV-status.

## INTRODUCTION

Tobacco smoking remains one of the main risk factors for the development of head and neck squamous cell carcinoma (HNSCC), including oropharyngeal squamous cell carcinoma (OPSCC). However, in the recent decade the incidence of human papilloma virus (HPV)-associated OPSCC has exceeded tobacco related OPSCC especially in Northern European countries [1–4].

Besides influencing the etiology of OPSCC, tobacco smoking also impacts treatment response and hence probability of survival for cases regardless of HPV-status [5–7]. Smoking is reported to have a higher impact on survival for cases with HPV-neg. tumours compared to cases with HPV-pos. tumours [8–11], although these findings were reached by evaluation of qualitative smoking status at diagnosis (current smoker, former smoker, and non-smoker) alone. The effect of accumulated smoking exposure in form of number of pack-years has not previously been considered in such evaluations.

The newly published TNM8 classification includes p16-status as a single marker in the staging of OPSCC. However, sole p16INK4A(p16)-overexpression has proven as an insufficient marker for HPV-positivity, as well as for predicting outcome [12–14]. Approximately 10–15% of all OPSCCs are p16-positive but HPV-DNA-negative and correspondingly have a significantly worse survival rate compared to HPV+/p16+ OPSCCs [13, 15]. Identification of both HPV-DNA and p16-status should preferably be included in cancer staging and in studies evaluating the impact of HPV-status.

The purpose of this study was to evaluate the impact of accumulated tobacco smoking exposure in form of number of pack-years on overall and progression-free survival (OS and PFS) for OPSCC cases in relation to HPV- and p16-status.

## RESULTS

We included 1316 cases; 75% (*n* = 993) from Eastern Denmark and 25% (*n* = 323) from Giessen (Table 1). Collectively, 48% (*n* = 629) of the cases were HPV-pos., with 56.9% being HPV-pos. from Denmark and 19.8% being HPV-pos. from Giessen (Table 1). Overall, the German case cohort presented with a more advanced stage and had a worse performance score in comparison to the Danish cohort. A larger proportion of the German cases were active smokers at the time of diagnosis. The median numbers of pack-years among smokers were 37 (range: 0–208) and 39 (range: 0–132) for the Danish and German cohort (*p* = 0.18), respectively. A higher proportion of cases with HPV-neg. tumours was smokers compared to cases with HPV-pos. tumours (95% vs. 68%, *p* < 0.001) (Table 1). The median number of pack-years among smokers was 30 (range: 0–208) and 42 (range: 0–147) for the cases with HPV-pos. and HPV-neg. tumours respectively (*p* < 0.001).

**Table 1:** Baseline characteristics of 1316 patients diagnosed in Denmark or Giessen, Germany, with HPV-positive or HPV-negative oropharyngeal squamous cell carcinoma, during the period 2000–2014

Variable	Total *n* (%)	Denmark *n* (%)	Giessen, Germany *n* (%)	*p*	HPV-neg. *n* (%)	HPV-pos. *n* (%)	*P*
	1316 (100.0)	993 (74.5)	323 (24.5)		687 (52.2)	629 (47.8)	
HPV-pos. (%)	629 (47.8)	565 (56.9)	64 (19.8)	< 0.01	-	-	
Patients from Giessen (%)	-	-	-		259 (37.7)	64 (10.2)	< 0.01
Male gender (%)	965 (73.3)	720 (72.5)	245 (75.9)	0.27	492 (71.6)	473 (75.2)	0.16
Median Age (IQR)	-	59.50 (53.53;66.08)	58.87 (52.57;64.90)	0.29	59.83 (54.05;66.35)	58.81 (52.47;65.27)	0.02
Median Year of Diagnosis (IQR)	-	2009 (2005;2012)	2005 (2002;2007)	< 0.01	2006 (2003;2009)	2009 (2005;2012)	< 0.01
Smokers (%)	1078 (81.9)	793 (79.9)	285 (88.2)	< 0.01	649 (94.5)	429 (68.2)	< 0.01
Pack-years							
Non-smoker	238 (18.1)	200 (20.1)	38 (11.8)		38 (5.5)	200 (31.8)	
< 20	241 (18.3)	191 (19.2)	50 (15.5)		86 (12.5)	155 (24.6)	
21–30	169 (12.8)	112 (11.3)	57 (17.6)		104 (15.1)	65 (10.3)	
> 30	668 (50.8)	490 (49.3)	178 (55.1)	< 0.01	459 (66.8)	209 (33.2)	< 0.01
Median number of pack-years among smokers [range]	-	37 [0;208]	39 [0;132]	0.18	42 [0;208]	30 [0;147]	< 0.01
*T*-stage							
T1	270 (20.5)	202 (20.3)	68 (21.1)		113 (16.4)	157 (25.0)	
T2	542 (41.2)	450 (45.3)	92 (28.5)		221 (32.2)	321 (51.0)	
T3	331 (25.2)	252 (25.4)	79 (24.5)		221 (32.2)	110 (17.5)	
T4	173 (13.1)	89 (9.0)	84 (26.0)	< 0.01	132 (19.2)	41 (6.5)	< 0.01
N-stage							
N0	291 (22.1)	202 (20.3)	89 (27.6)		202 (29.4)	89 (14.1)	
N1	575 (43.7)	532 (53.6)	43 (13.3)		148 (21.5)	427 (67.9)	
N2	335 (25.5)	158 (15.9)	177 (54.8)		248 (36.1)	87 (13.8)	
N3	115 (8.7)	101 (10.2)	14 (4.3)	< 0.01	89 (13.0)	26 (4.1)	< 0.01
UICC8-stage							
I	493 (37.5)	441 (44.4)	52 (16.1)		80 (11.6)	413 (65.7)	
II	251 (19.1)	190 (19.1)	61 (18.9)		107 (15.6)	144 (22.9)	
III	205 (15.6)	156 (15.7)	49 (15.2)		146 (21.3)	59 (9.4)	
IV	367 (27.9)	206 (20.7)	161 (49.8)	< 0.01	354 (51.5)	13 (2.1)	< 0.01
Performance score							
0	711 (54.0)	692 (69.7)	19 (5.9)		229 (33.3)	482 (76.6)	
1	436 (33.1)	240 (24.2)	196 (60.7)		319 (46.4)	117 (18.6)	
2+	169 (12.8)	61 (6.1)	108 (33.4)	< 0.01	139 (20.2)	30 (4.8)	< 0.01

The main parameter contributing to missing data was performance score (missing in 18.9% cases). Further, data on number of pack-years, smoking status, and N-stage were missing for 7.9%, 2.2%, 0.2% of the cases.

**Figure 1:**
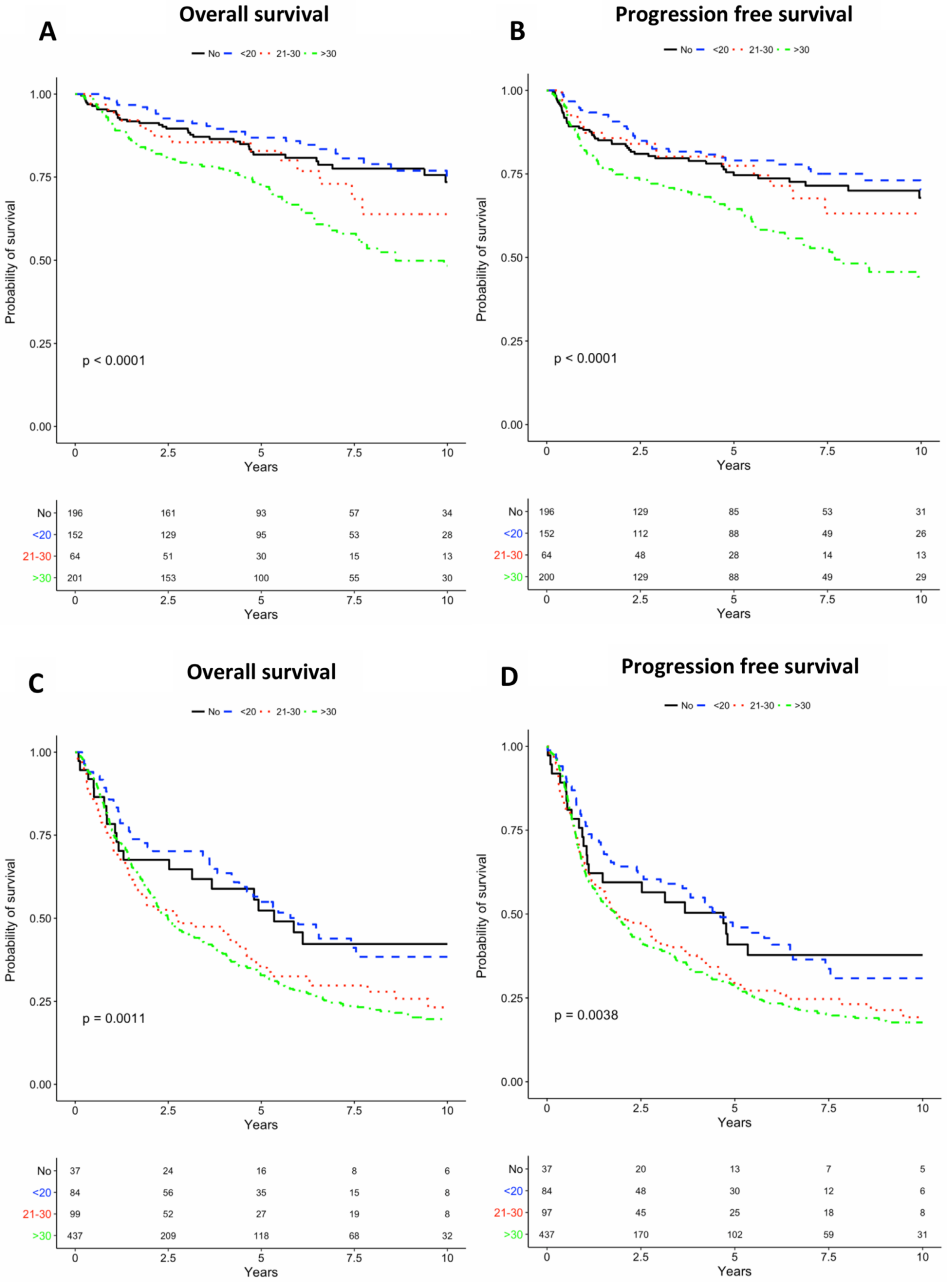
Kaplan-Meier (KM) curves for overall survival (OS) and progression-free survival (PFS) are presented below. (**A**) KM-curves for OS for HPV positive stratified by smoking. (**B**) KM-curves for PFS for HPV positive stratified by smoking. (**C**) KM-curves for OS for HPV negative stratified by smoking (**D**) KM-curves for PFS for HPV negative stratified by smoking.

The median follow-up time for OS was 5.23 years (range: 0.2–10.0 years) for the cases with HPV-pos. tumours and 2.64 years (range: 0.0–10.0 years) for cases with HPV-neg. tumours. In the unadjusted analysis cases with HPV-pos. tumours had better OS and PFS compared with cases with HPV-neg. tumours (log-rank *p* < 0.0001). The 2-year OS was 89.3% and 59.5%, and the PFS was 82.7% and 50.5% for cases with HPV-pos. and HPV-neg. tumours respectively. OS and PFS based on number of pack-years stratified on HPV-status showed decreasing survival probability with increasing number of pack-years regardless of HPV-status (Figure 1).

**Figure 2:**
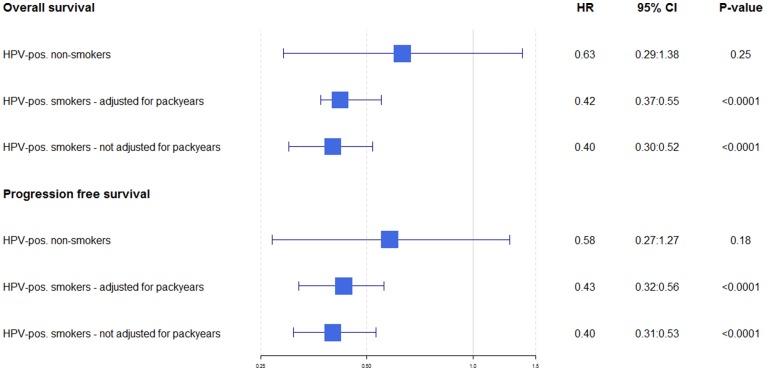
Hazard ratios for overall and progression free survival when HPV-positive cases are compared directly to HPV-negative. For both OS and PFS, HPV-positive cases are plottet (compared to HPV-negative; in the first row the unadjusted HRs are shown, the second row smokers adjusted for number of pack-years, and in the third row smokers not adjusted for pack-years.

Smokers (i.e. current or former smokers) had a non-significant elevated risk of death and progression compared to non-smokers regardless of HPV-status, however the difference appeared to be bigger for the cases with HPV-neg. tumours (Table 2). When analyzing the impact of HPV-status for non-smokers and smokers, we found that the difference in OS and PFS between non-smoking cases with HPV-pos. tumours and cases with HPV-neg. tumours were not significant. There was however, a highly significant difference for smokers for both OS and PFS (Figure 2).

**Table 2:** Impact of smoking on overall survival (OS) and progression free survival (PFS) for HPV-positive and HPV-negative oropharyngeal squamous cell carcinoma patients, adjusted for age, sex, HPV-status, year of diagnosis, T-stage, N-stage, overall stage, performance score and cohort

	OS - HR (95%CI)	*P*	PFS - HR (95%CI)	*P*
HPV-neg. patients				
Average smoker vs. non-smoker	1.48 (0.94, 2.32)	0.09	1.36 (0.88, 2.12)	0.17
Effect of 10 additional pack-years	1.05 (1.02, 1.09)	< 0.01	1.05 (1.02, 1.09)	< 0.01
Pack-years				
Non-smoker	1 (Ref)		1 (Ref)	
< 20	0.94 (0.55, 1.60)	0.81	0.91 (0.55, 1.50)	0.72
21–30	1.65 (1.00, 2.70)	0.05	1.53 (0.94, 2.50)	0.09
30	1.69 (1.07, 2.70)	< 0.05	1.56 (1.00, 2.40)	0.05
HPV-pos.				
Average smoker vs. non-smoker	1.24 (0.86, 1.77)	0.25	1.24 (0.89, 1.72)	0.21
Effect of 10 additional pack-years	1.09 (1.02, 1.16)	< 0.01	1.09 (1.02, 1.15)	< 0.01
Pack-years				
Non-smoker	1 (Ref)		1 (Ref)	
20	0.77 (0.48, 1.30)	0.29	0.79 (0.51, 1.20)	0.29
21–30	1.02 (0.57, 1.80)	0.96	0.86 (0.49, 1.50)	0.60
30	1.58 (1.08, 2.30)	< 0.05	1.50 (1.1, 2.20)	< 0.01

In analysis adjusted for age, sex, HPV-status, year of diagnosis, *T*-stage, *N*-stage, overall stage, performance score and cohort (Eastern DK vs. Giessen), cases with HPV-neg. tumours demonstrated a significantly higher hazard rate in overall survival (OS-HR) in the groups of 21–30 pack-years (OS-HR: 1.65 (95%CI 1.0;2.7)) and > 30 pack-years (OS-HR: 1.69 (95%CI 1.1;2.7)) compared to non-smokers, whereas for the cases with HPV-pos. tumours only the risk estimate for the group of > 30 pack-years compared with non-smokers (OS-HR: 1.58 (95%CI 1.08;2.3)) reached statistical significance (Table 2). The HRs for cases with HPV-pos. and HPV-neg. tumors were not significantly different. When analyzing the difference between the Danish and the German cohort, there were no significant difference, however there was a tendency towards a greater impact of pack-years for the Danish cohort (Supplementary Table 1).

**Figure 3:**
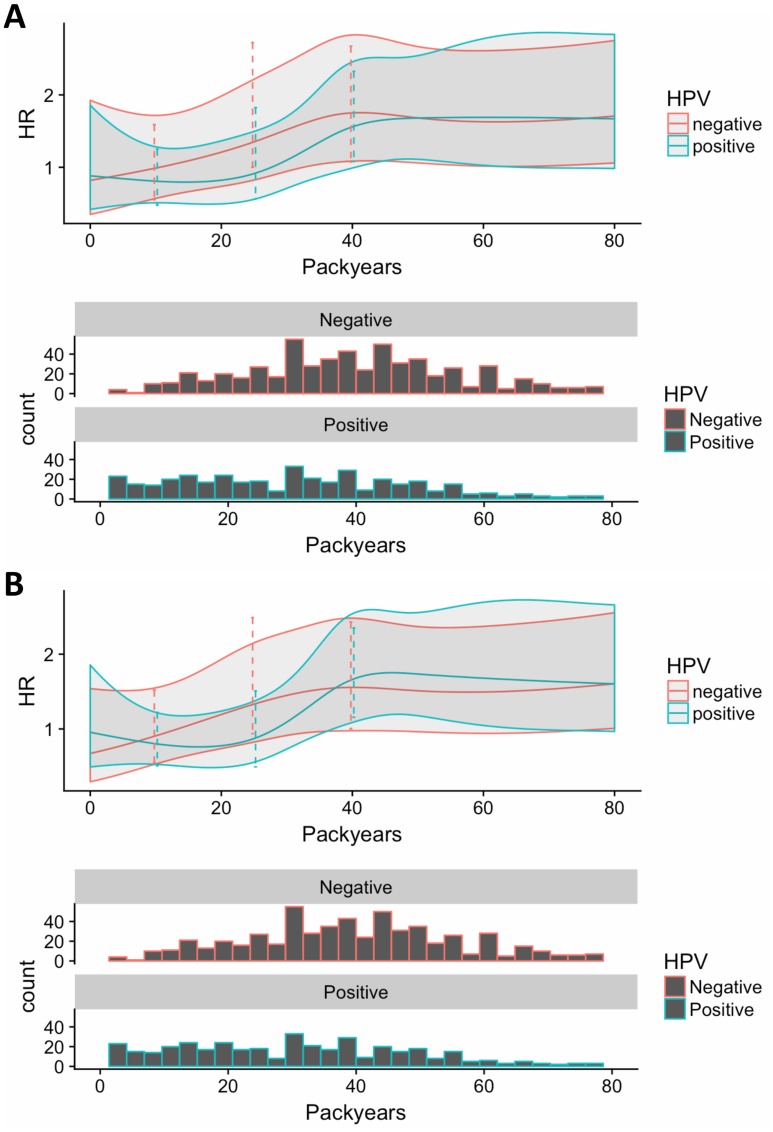
Continuous smoking exposure for (**A**) overall survival and (**B**) progression free survival. Here, packyears are included through a restricted cubic spline. We have set non-smokers packyears equal to the median packyears among smokers, while also including an indicator variable corresponding to smoking yes/no. This implies that the estimate for the smoking variable corresponds to comparing a smoker with a median number of packyears with a non-smoker (instead of a smoker with 0 packyears with a non-smoker, if packyears are set to 0 for non-smokers). In addition, we scale packyears by 10, i.e. effects are per 10 packyears. Finally, we allow the effect of smoking as well as the effect of pack-years to depend on HPV-status. The impact of packyears (and smoking status).

Considering smoking exposure as a continuous variable an additional 10 pack-years of smoking increased the risk of death and progression statistically significantly for both cases with HPV-pos. tumours (OS-HR: 1.09, PFS-HR: 1.09) and cases with HPV-neg. tumours (OS-HR: 1.05, PFS-HR: 1.05), although with no statistically significant difference between the two groups (Table 2). Considering the non-linear model in the restricted cubic spline, there was a slight tendency to a larger negative impact of smoking for cases with HPV-neg. tumours compared to cases with HPV-pos. tumours with low numbers of pack-years, however with high numbers of pack-years the survival was similar for the two groups (Figure 3). Furthermore, there was a tendency that the hazard ratio did not increase further with more than 40 pack-years. There was no overall significant difference between cases with HPV-pos. tumours and cases with HPV-neg. tumours in the non-linear model.

## DISCUSSION

In this study including 1316 cases from both high and low HPV-prevalent areas, we identified that smoking status at diagnosis had a stronger negative impact on survival for cases with HPV-neg. tumours compared to cases with HPV-pos. tumours, however not statistically significant. This is compatible with other reports [8–11]. However, smoking status at diagnosis is not necessarily the paramount parameter for evaluating the impact of smoking, as smokers (i.e. former or current) may have dissimilar exposure patterns of tobacco smoking. It remains questionable if the status of a former smoker should be categorized solely or grouped with either non-smokers or current smokers. These cases may have diverse smoking exposure and therefore collective evaluation is challenging. Further, the reliability of cases’ recollection of smoking habits, e.g. recall bias, may be an important source of bias.

For this reason, we also included analysis on accumulated smoking exposure in forms of pack-years. This analysis revealed that overall, the number of pack-years did not have a statistically significantly different impact on survival between cases with HPV-pos. and cases with HPV-neg. tumours. We identified a tendency towards a more pronounced effect on survival for cases with HPV-neg. tumours with low numbers of pack-years compared to cases with HPV-pos. tumours. This difference evened out with increasing numbers of pack-years, and further, the increase in HRs with pack-years plateaued for more than 40 pack-years according to the non-linear model. This might be explained by the higher risk of death from other diseases with high numbers of pack-years.

Considering former reports on differences on impact of smoking between cases with HPV-pos. and HPV-neg. tumours as mentioned previously, it is remarkable that the analysis of cumulative smoking exposure yielded no overall significant difference. However, it might be explained by the difference in impact, which is only present at low numbers of pack-years, or limited power among cases with HPV-pos. tumours.

It should be noted that only very few HPV-negative cases were non-smokers (~5%). This might explain why we did not find a significant difference in the impact of smoking status between cases with HPV-pos. tumours and cases with HPV-neg. tumours, and maybe also why we did not find a significant impact of HPV-status among non-smokers.

Time-to-treatment initiation (TTI) and cause-of-death (COD) data are important information; in the Danish cohort, the TTI has been reduced yearly (now less than 30 days). It is reported that TTI>60 days affected both overall survival and PFS most evident in the HPV-negative group of cases [16]. COD has also been reported in the Danish group; at follow-up 723 (47.5%) patients were deceased. For these cases, the median time to and cause of death were determined: oropharyngeal cancer (*n* = 432; 1.00 year), secondary malignancies (*n* = 131; 2.37 years), cardiovascular and pulmonary causes (*n* = 58; 3.48 years), and unspecified causes (*n* = 102; 3.42 years) [17]. It should be noted that the difference in follow-up times for the two cohorts is due to the difference in mortality for the two cohorts, e.g. the Giessen cohort has a significant higher mortality and hence a shorter follow-up.

The inclusion of cases from different geographical locations is an advantage of our study, especially considering the difference in HPV-prevalence and smoking habits. It warrants that the results are applicable to other centers. Although cumulative smoking exposure is a more precise measure for evaluating the impact of smoking, it should be noted that this data is obtained retrospectively and therefore underlies bias. A study by Gillison et al. showed that smoking during treatment had an significant negative impact on outcome, independent of HPV-status [5]. Unfortunately, our groups do not have data on smoking-habits following treatment. A notable limitation to this study is the relative low numbers of no-smokers compared to smokers. This will minimize the power of comparing non-smokers to smokers.

In conclusion, analyzing 1316 cases from Eastern Denmark and Giessen, Germany in the period from 2000 to 2014, we found that smoking-status at the time of diagnosis and adding an additional 10 pack-years of smoking significantly impacts treatment response independently of HPV status. This study underlines that tobacco smoking influences survival, and patients despite age should be encouraged to prioritize smoking cessation. The survival impact between low-smokers and none-smokers remains however unclear although this study suggests that the difference is very small. Likewise, data and clinical understanding is sparse in the none-virus, none-smoking induced head and neck squamous cell carcinomas group.

## MATERIALS AND METHODS

Case data included in this study was collected retrospectively from two consecutive population-based Danish and German cohorts. The Danish cohort consists of cases diagnosed and/or treated with OPSCC at the University Hospital of Copenhagen, Rigshospitalet or University Hospital of Copenhagen, Herlev, from 2000 to 2014. The University Hospital of Copenhagen covers cases from the Eastern Denmark region, This region comprises 46% of the approximately 5.5 million inhabitants in Denmark. The Danish healthcare system provides the population with free access to all diagnostics and treatments from general practitioners to hospitals, financed by general taxes. This means that cases were not selected, and treatment was initiated when indicated, irrespective of e.g. case economy and insurance. Cases were identified through the DAHANCA (Danish Head and Neck Cancer group) database and validated through the national Danish Pathology Data Bank (DPDB). The German cohort consists of all cases diagnosed with OPSCC at the Department of Otorhinolaryngology, Head and Neck Surgery, at the University of Giessen from 2000 to 2009. Cases were prospectively recorded by the Giessen cancer registry database.

All tumours were evaluated for p16-overexpression, and considered positive if expression was above 70% in both cytoplasm and nucleus [18]. Furthermore, all tumours were examined for HPV-DNA by polymerase chain reaction [3, 4, 19]. Tumours with a double positivity for HPV-DNA and p16 were defined as HPV-positive. All other combinations of HPV-DNA- and p16-status were defined as HPV-negative.

Date of diagnosis was registered as the date of diagnostic verification based on biopsy specimens. Cases alive at last follow-up date were censored. Overall survival (OS) was calculated as time from date of diagnosis until death from any cause or censoring. Progression free survival (PFS) was calculated as time from date of diagnosis until progression, death from any cause, or censoring.

We included data on gender, age at diagnosis, smoking status (non-smoker vs smoker (current or former)), pack-years at diagnosis, TNM-stage, and ECOG performance score. All cases were restaged to the 8th edition of UICC/AJCC staging. N2a, N2b and N2c were merged to N2. This information was retrospectively obtained by evaluating the medical charts of the cases.

### Statistical analysis

OS and PFS were illustrated by means of the Kaplan-Meier estimator stratified by 1) HPV/p16 status and 2) pack-years. Hazard ratios (HR) and 95% confidence intervals (CI) in relation to number of pack-years were estimated with Cox proportional hazard regression for OS and PFS, respectively. Pack-years were parameterized in two versions: 1) as a categorical variable (never-smoker, <=20 pack-years, 21–30 pack-years,> 30 pack-years) and 2) as a continuous variable included in the model while also adjusting for ever-smoking. Non-linearity was tested using a restricted cubic spline. Separate effects for cases with HPV-pos. tumours. and cases with HPV-neg. tumours were estimated. The analyses were adjusted for age, sex, HPV-status, year of diagnosis, T-stage, N-stage, overall stage, performance score and cohort (Eastern DK vs. Giessen).

All analyses were based on complete cases and we applied a 5% significance level. R version 3.3.3 using packages rms and survival was used for all analyses [20].

## SUPPLEMENTARY MATERIALS


