# Supplementary material for: Impact on survival of tobacco smoking for cases with oropharyngeal squamous cell carcinoma and known human papillomavirus and p16-status: a multicenter retrospective study
Source: Oncotarget. 2019 Jul 23;10(45):4655–63. doi: 10.18632/oncotarget.27079 (PMC6659794; doi:10.18632/oncotarget.27079)
Supplement: Supplementary file 1 [file oncotarget-10-4655-s001.pdf]

Impact on survival of tobacco smoking for cases with oropharyngeal squamous cell carcinoma and known human papillomavirus and p16-status: a multicenter retrospective study

SUPPLEMENTARY MATERIALS

Supplementary Table 1: Impact of smoking on overall survival (OS) and progression free survival (PFS) for the Danish and the German cohort, adjusted for age, sex, HPV-status, year of diagnosis, T-stage, N-stage, overall stage, and performance score

	The Danish cohort	The German cohort	*P*
OS - HR (95%CI)			
Non-smoker	1 (Ref)	1 (Ref)	
< 20	0.92 (0.61, 1.39)	0.64 (0.33, 1.22)	
21–30	1.40 (0.92, 2.14)	1.20 (0.67, 1.22)	
30	1.79 (1.28, 2.52)	1.08 (0.64, 1.81)	0.26
effect of 10 additional pack-years	1.06 (1.02, 1.10)	1.05 (1.00, 1.12)	0.83
PFS - HR (95%CI)			
Non-smoker	1 (Ref)	1 (Ref)	
20	0.91 (0.62, 1.34)	0.67 (0.37, 1.23)	
21–30	1.32 (0.88, 1.97)	1.09 (0.63, 1.88)	
30	1.73 (1.26, 2.38)	1.06 (0.65, 1.74)	0.30
effect of 10 additional pack-years	1.07 (1.03, 1.11)	1.05 (1.00, 1.11)	0.65
